# Evidence for Different Pathways during Horizontal Gene Transfer in Competent *Bacillus subtilis* Cells

**DOI:** 10.1371/journal.pgen.1000630

**Published:** 2009-09-04

**Authors:** Dawit Kidane, Begoña Carrasco, Candela Manfredi, Katharina Rothmaier, Silvia Ayora, Serkalem Tadesse, Juan C. Alonso, Peter L. Graumann

**Affiliations:** 1 Mikrobiologie, Fakultät für Biologie, Universität Freiburg, Freiburg, Germany; 2 Department of Microbial Biotechnology, Centro Nacional de Biotecnología, CSIC, Campus Universidad Autónoma de Madrid, Madrid, Spain; Université Paris Descartes, INSERM U571, France

## Abstract

Cytological and genetic evidence suggests that the *Bacillus subtilis* DNA uptake machinery localizes at a single cell pole and takes up single-stranded (ss) DNA. The integration of homologous donor DNA into the recipient chromosome requires RecA, while plasmid establishment, which is independent of RecA, requires at least RecO and RecU. RecA and RecN colocalize at the polar DNA uptake machinery, from which RecA forms filamentous structures, termed threads, in the presence of chromosomal DNA. We show that the transformation of chromosomal and of plasmid DNA follows distinct pathways. In the absence of DNA, RecU accumulated at a single cell pole in competent cells, dependent on RecA. Upon addition of any kind of DNA, RecA formed highly dynamic thread structures, which rapidly grew and shrank, and RecU dissipated from the pole. RecO visibly accumulated at the cell pole only upon addition of plasmid DNA, and, to a lesser degree, of phage DNA, but not of chromosomal DNA. RecO accumulation was weakly influenced by RecN, but not by RecA. RecO annealed ssDNA complexed with SsbA *in vitro*, independent of any nucleotide cofactor. The DNA end-joining Ku protein was also found to play a role in viral and plasmid transformation. On the other hand, transfection with SPP1 phage DNA required functions from both chromosomal and plasmid transformation pathways. The findings show that competent bacterial cells possess a dynamic DNA recombination machinery that responds in a differential manner depending if entering DNA shows homology with recipient DNA or has self-annealing potential. Transformation with chromosomal DNA only requires RecA, which forms dynamic filamentous structures that may mediate homology search and DNA strand invasion. Establishment of circular plasmid DNA requires accumulation of RecO at the competence pole, most likely mediating single-strand annealing, and RecU, which possibly down-regulates RecA. Transfection with SPP1 viral DNA follows an intermediate route that contains functions from both chromosomal and plasmid transformation pathways.

## Author Summary

Many bacteria can actively acquire novel genetic material from their environment, which leads to the rapid spreading of, for example, antibiotic resistance genes. The bacterium *Bacillus subtilis* can differentiate into the state of competence, in which cells take up ssDNA through a DNA uptake complex that is specifically localized at a single cell pole. DNA can be integrated into the chromosome, via RecA, or can be reconstituted as circular dsDNA, if derived from plasmid or from viral DNA. We show that RecO, RecU, and Ku proteins, but not RecA, are important for plasmid transformation, and differentially accumulate at the polar DNA uptake machinery. Upon addition of any kind of DNA, the assembly of RecU at the competence pole dissipated, while RecA formed filamentous structures that rapidly grew and shrank within a 1 minute time scale. RecO visibly accumulated at the competence machinery only upon addition of plasmid DNA, but not of chromosomal DNA. *In vitro*, RecO was highly efficient at enhancing the annealing of complementary strands covered by SsbA, without the need for any nucleotide cofactor. The findings show that competent cells possess a dynamic recombination machinery and provide visual evidence for the existence of different pathways for transformation with chromosomal DNA or with plasmid DNA.

## Introduction

Natural genetic transformation is an efficient mechanism of horizontal gene transfer between bacteria, and thus of the acquisition of novel genetic material. At the onset of stationary phase, *Bacillus subtilis* cells can become competent (up to 20% of all cells), specified through the induction of proteins mediating the binding of environmental DNA to the cell surface, and upon its processing, transporting the single-stranded (ss) DNA into the cytosol [1],[2]. Recombination proteins integrate homologous or “*partially*” homologous foreign DNA into the chromosome, or allow the establishment of autonomously replicating molecules (plasmid or viral DNA). The competence-specific DNA-uptake proteins and a large set of additional proteins are under the control of the master competence transcription factor, ComK [3],[4]. DNA uptake occurs in a highly processive manner [5] at a single cell pole, as exemplified by visualization of ComGA and ComFA, two presumed ATPases involved in DNA translocation [6],[7]. Environmental double-stranded (ds) DNA somehow crosses the cell wall to bind to the ComEA membrane protein, and is nicked (and thus fragmented) by the NucA endonuclease. Then, one strand is transported across the membrane via ComEC, whereas the other strand is degraded to nucleosides outside the cell [1],[2]. Hence, the DNA uptake machinery takes up linear ssDNA molecules, which are then available to the intracellular recombination machinery.

More than 12 genes have been shown to be involved in genetic recombination in *B. subtilis*. The absence of RecA, the central recombination protein that mediates strand invasion between homologous linear ssDNA and supercoiled dsDNA (forming D-loop intermediates) and strand exchange, leads to a reduction in transformation with chromosomal DNA by more than 4 orders of magnitude [8],[9]. A defect in any gene classified within the α (*recF*, *recO* and *recR*), β (*addA* and *addB*), γ (*recH* and *recP*), δ (*recN*), ε (*recU*) or ζ (*recJ*, *recS* and *recQ*) epistatic group reduces the frequency of chromosomal transformation only 4-fold or less in an otherwise wild type (wt) background [10],[11], suggesting that alternative pathways may help or modulate RecA to achieve this process. This conclusion is in agreement with *in vivo* data showing that in the simultaneous absence of RecA modulators (i.e. in a *addAB recO* double mutant strain), chromosomal and plasmid transformation are blocked [12]. On the other hand, the transformation frequency with plasmid DNA is reduced more than 20-fold in *recO* or in *recU* mutant cells, but is only moderately (less than 4-fold) reduced in all other *rec* mutants tested, and is not at all reduced in *recA* mutant cells [12],[13],[14]. These data suggest that a redundancy of factors involved in transformation and different pathways exist during transformation [11]. However, the use of different *B. subtilis* genetic backgrounds and/or of different DNA substrates in the past has complicated the interpretation of genetic data.

**Figure 1:**
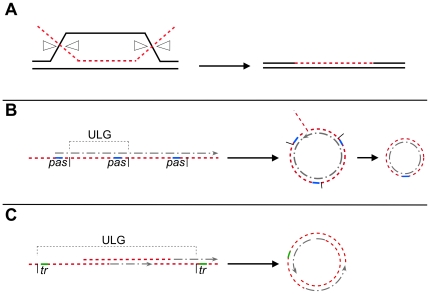
Model for natural transformation with different kinds of DNAs. (A) With chromosomal transformation, the incoming ssDNA (red line) displaces the identical strand in the duplex of chromosomal DNA and pairs with the complementary strand. The D-loop structure is resolved as indicated by white triangles, and the displaced DNA strand degraded. The ends are sealed and one of the daughter cell inherits the donor DNA; (B), with oligomeric plasmid DNA, the incoming ssDNA (red line) is longer than a unit-length plasmid genome (ULG), indicated by vertical bars. Replication initiated at the primosome assembly site (*pas*, indicated as a blue line), may convert the ssDNA onto dsDNA. After DNA replication, homologous regions must recombine to generate a circular intermediate that is then converted into a single plasmid monomer. (C) With viral DNA, the fragmented viral ssDNA (red line) recombines intermolecularly to generate a ULG after filling-in the gaps. The assembled dsDNA must recombine intramolecularly, probably through the terminal redundancy (tr, depicted in green) to generate a circular molecule.

Three different pathways for transformation have been proposed. Firstly, if taken up DNA contains sufficient homology to the chromosome (<than 20% divergence in several hundred bp), ssDNA may be directly incorporated into the chromosome via intermolecular recombination (chromosomal transformation) [15], setting up heteroduplex DNA (with one parental DNA strand getting degraded) (Figure 1A). Upon re-entering into the vegetative state, one daughter cell receives a chromosome copy derived from the incorporated DNA, and may thus become transformed, while the other cell receives the parental DNA. Secondly, if taken up ssDNA lacks homology with the chromosome it must be annealed to form a circular dsDNA molecule (Figure 1B) and be self-replicative (i.e. contain an origin of replication). Interestingly, only multimeric, but not monomeric, plasmid DNA can lead to transformation [14],[16]. A third scenario may occur in case of viral transfection. It has been shown that the average length of the entering ssDNA is around 12-kb (reviewed by [17]), while phage SPP1 is composed of 45.5-kb DNA. Therefore, in order to form a full-length phage dsDNA molecule from incoming ssDNA fragments, these must be recombined intermolecularly to yield a partially annealed full-length molecule which can then be replicated (Figure 1C). The terminal repeats may be recombined intramolecularly to form a circular molecule that can be proficient for replication. In the absence of homology with recipient DNA or of an autonomous replication unit, taken up DNA is degraded [17].

The visualization of proteins involved in competence in *B. subtilis* has recently provided a new tool to study transformation in time and space. During the state of competence, the DNA uptake machinery, SsbB (also termed YwpH), DprA (Smf), CoiA (YjbF) [6],[18], and RecA, colocalizes at a single cell pole, whereas RecN oscillates between the poles [19]. Internalized linear ssDNA appears to stop the oscillation of RecN, which is a ssDNA-binding protein [20],[21], and DprA is important for loading of RecA to incoming ssDNA [22]. Thread-like structures of RecA are observed to emanate from the competence pole [19]. These have been proposed to guide ssDNA onto the nucleoid that contains the chromosome, in order to mediate strand exchange with the recipient DNA. In support of this idea, a non-functional RFP-RecA fusion, which accumulates at the competence pole, but fails to form threads after addition of DNA, is entirely deficient in transformation [19]. Therefore, transformation with DNA appears to be a spatially highly organized process.

Genetic data suggest that RecO and RecU (analogue of *Escherichia coli* RuvC) are not essential during chromosomal transformation, and their significant role played during plasmid transformation is poorly understood [15]. Both RecO and RecU have an important function during DNA double strand break (DSB) repair [21],[23],[24],[25],[26]. Here, RecO promotes loading of RecA onto SsbA-coated ssDNA, and RecU catalyzes the resolution of HJs [27],[28]. To verify the genetic requirements during transformation, we analyzed the relevance of RecA, RecO, and RecU and other proteins in transformation with three different substrates, chromosomal, plasmid and viral DNA, within a single genetic background. We also investigated the involvement of Ku protein, which is involved in DNA end joining during DSB repair [29]. We show that RecO, RecA and RecU proteins localize dynamically and differentially at the competence pole, dependent on the DNA substrate added to the cells, and in accordance with this, that the proteins perform different roles in promoting recombination of incoming DNA, which also depends on the nature of incoming DNA. Our work shows that recombination proteins appear at different time points and steps during DSB repair and transformation, showing that they obtain different functional specificities during the two processes.

## Results

### RecO, RecU, and Ku play an important role during plasmid or viral DNA transformation

Transformation efficiencies have been assayed for different types of DNA and in various different *B. subtilis* strains, all of which carried inducible prophages, which complicates all analyses [10]. We therefore investigated the function of *B. subtilis* DNA recombination and repair proteins during transformation in the same phage-free background (strain BG214 lacks the ICE*Bs*1 transposon and prophage SPβ, and PBSX cannot be induced), and with three different DNA substrates (chromosomal, plasmid and viral DNA) in parallel. Previously, the transformation defects of Δ*recA*, Δ*recO*, Δ*recR*, *recF*15, Δ*recU* and Δ*recN* cells have been analysed using different conditions (replicons, markers, DNA concentrations, etc.) or viral transfection (also termed viral transformation) (reviewed in [11]), but were re-evaluated here for a direct comparison. The assays were normalized to actual DNA uptake and to cell viability (see Material and Methods). The selected DNA substrates allowed us to study presumably different recombination events: (i) recombination of internalized chromosomal ssDNA with the host chromosome based on the existence of homology with recipient DNA (intermolecular recombination, Figure 1A), (ii) conversion of internalized ssDNA to dsDNA and circularization by intramolecular recombination in the case of replicative plasmid DNA (Figure 1B), or (iii) internalization of fragmented (i.e. less than unit-length) viral SPP1 ssDNA, which has to be converted to dsDNA, recombined to generate full-length viral DNA, and circularized (both, inter- and intramolecular recombination) (Figure 1C).

The frequency of appearance of chromosomal transformants was ∼10,000-fold decreased in the Δ*recA* strain, whereas chromosomal transformation efficiency in the Δ*recO*, Δ*recR*, *recF*15, Δ*recU*, *recU*71, Δ*ruvAB*, Δ*recN* or Δ*ku* (also termed Δ*ykoV*) deficient strains did not change more than 2- to 3-fold relative to the wild type strain (Table 1). These results reinforce the idea that RecA is required for intermolecular recombination, and show that presynaptic (RecN, RecF, RecO, or RecR) or postsynaptic (RecU and RuvAB) functions are not required for this pathway in an otherwise wt background, or may have redundant roles in chromosomal transformation, because negative effects are seen with some double mutations [10].

**Table 1:** RecO, RecU, and Ku play an important role in plasmid transformation, but are dispensable for transformation with chromosomal DNA.

Relevant genotype	Normalized plasmid transformation	Normalized SPP1 transfection	Normalized chromosomal transformation
wt	100 (1.5×10^4^)	100 (2.8×10^4^)	100 (5.5×10^6^)
Δ*recO*	3.1	2.8	54
*recF*15	86	120	74
Δ*recR*	72	100	69
Δ*recU*	^3.8^	3.9	^53^
*recU*71	2.9	3.3	41
Δ*ruvAB*	23	60	82
Δ*recN*	55	51	72
Δ*ku* (*ykoV*)	17	19	56
Δ*recA*	98	1.4	<0.01

Competent *B. subtilis* cells auxotrophic for methionine were transformed with chromosomal DNA from a *met* ^+^ (SB19) strain. The yield of *met* ^+^ transformants (chromosomal transformation), kanamycin-resistant transformants (plasmid pUB110 transformation), and SPP1 transfection was corrected for DNA uptake and cell viability and the values obtained normalized relative to that of the *rec* ^+^ strain, taken as 100 (between parentheses, the number of transformants/transfectants obtained per 0.1 µg DNA/ml). The results are the average of at least five independent experiments and are within a 10% standard error.

The efficiency of plasmid transformation was not affected in the absence of RecA (Table 1). Plasmid establishment was marginally impaired in *recF*15, Δ*recR* or Δ*recN* competent cells (2-fold), and slightly impaired (∼4-fold) in Δ*ruvAB* cells compared with wild type cells (Table 1). However, plasmid establishment was reduced ∼6-fold in Δ*ku* cells and 25- to 35-fold in Δ*recO*, *recU*71 or Δ*recU* cells (Table 1). These results show that RecO and RecU are required for plasmid transformation and that Ku plays a minor role in this pathway, whereas other presynaptic proteins (RecN, RecF and RecR) are not required in an otherwise wild type background. The defect seen in Δ*ruvAB* cells may be caused by a reduction of the resolution of HJs, but RecU seems to play an additional role in plasmid transformation, because *recU* mutant cells are 5-times more deficient in plasmid transformation than *ruvAB* mutants. This is consistent with the observation that the *rec*U71 strain, which encodes a RecU variant (RecU R71A) proficient in strand annealing and HJ resolution, but deficient in RecA modulation [30], is as defective in plasmid transformation as a *recU* deletion strain (Table 1). We therefore argue that the main function of RecU during plasmid transformation is not HJ resolution.

It has been shown that the average length of incoming donor ssDNA is ∼12-kb [17]. Mature SPP1 DNA is a linear 45.5-kb long dsDNA molecule with 4% of terminal redundancy [31], hence, internalized SPP1 ssDNA is fragmented into 3 or more pieces by the DNA uptake machinery. Intracellular reconstitution of SPP1 DNA may therefore involve both intermolecular recombination to reconstitute a full-length molecule, and intramolecular recombination to achieve circularization [15]. Consistent with this, transfection of SPP1 DNA was affected in recombination mutants involved in both chromosomal (*recA*) and plasmid transformation (*recU*, *recO* and *ku*). The frequency of SPP1 transfection was reduced ∼100-fold in Δ*recA*, ∼35-fold in Δ*recO*, ∼30-fold in *recU*71, ∼25-fold in Δ*recU*, or ∼5-fold in Δ*ku* cells (Table 1). SPP1 transfection was marginally impaired in Δ*recN* or Δ*ruvAB* competent cells (less than 2-fold relative to the *rec* ^+^ value) or not reduced in Δ*recR* or *recF*15 competent cells (Table 1).

These results demonstrate that: (i) RecA-mediated strand exchange is required for intermolecular recombination during chromosomal transformation, rather than for protection of incoming ssDNA from nuclease attack, (ii) RecO, RecU and to some extent Ku are involved in plasmid transformation, and (iii) phage transfection follows a route comprising viral- and host-encoded functions from both pathways.

### RecA threads are highly dynamic and form after addition of any kind of DNA

**Figure 2:**
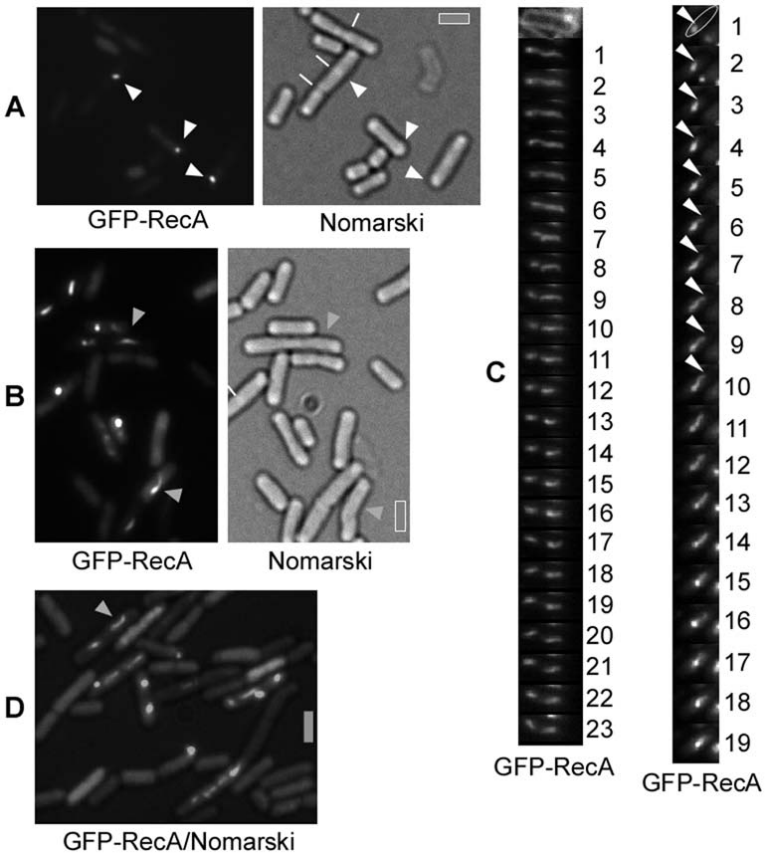
Fluorescence microscopy of GFP-RecA in cells grown to competence. (A) GFP-RecA forms polar foci in wt competent cells, and (B) threads after addition of chromosomal DNA. (C) Time-lapse with 1 min intervals of cells containing a GFP-RecA thread. Left panel: the upper image shows the outline of the cell through a membrane stain. The lower panels show an extended GFP-RecA thread that changes its shape every minute. Right panel: nucleation and extension of a RecA thread within one cell. In the upper image, a cell contains a GFP-RecA focus from which a thread extends for several minutes, as indicated by the white triangle. The thread appears to retract after minute 15, although it is unclear if this is based on true retraction or bleaching. (D) GFP-RecA threads after addition of supercoiled plasmid DNA. White triangles indicate polar foci, and grey triangles threads. Septa between cells are indicated in (A) by white lines. Grey bars 2 µm.

In cells grown to competence, a functional GFP-RecA fusion (see Material and Methods) colocalizes with competence ComGA protein to a single cell pole in ∼20% of all cells (this is the fraction of competent cells), or is associated with the nucleoids in the remaining cells [19] (Figure 2A). Addition of chromosomal DNA to competent cells leads to the formation of filamentous GFP-RecA structures, termed threads, which are very variable in length and shape (Figure 2B).

To investigate whether the threads are dynamic structures, we performed time-lapse microscopy, capturing images of cells grown to competence 10 to 30 min after addition of DNA within 1 min time intervals. Figure 2C and Videos S1, S2, S3, and S4 show examples of such experiments. A GFP-RecA thread, which changes its shape within each 1 min time interval, can be seen to extend from a single cell pole into the cell (Figure 2C) (left panel). At min 8, two apparently separate structures arise, one close to the cell pole and the other extending away from the competence cell pole. In all of the 26 movies taken, GFP-RecA threads showed highly dynamic localization, arising at one cell pole and extending into the cytosol, but never reaching the other cell pole. We have also observed discrete GFP-RecA foci that rapidly and continuously (for at least 20 min) moved through the cells (data not shown), but the nature of these assemblies is unclear. In Figure 2C, right panel, extension of a thread from a single focus can be seen to occur between min 2 to min 9, with peak extension between min 4 and 5. We were able to only capture 3 of such extension events from a single focus (with>300 cells analyzed), indicating that extension from the pole occurs very rapidly. In Video S4, GFP-RecA threads can also be seen to rapidly grow as well as shrink between 1 min intervals. Maximum extension of 0.6 (±0.2) µm/min was measured in 6 time lapse series, which is similar to the observed spreading of *E. coli* RecA onto dsDNA *in vitro* [32]. These experiments reveal rapid growth and shrinkage of RecA threads *in vivo*, and reinforce the idea that RecA threads guide incoming ssDNA from the pole onto nucleoids for homology search, ensuring maximum efficiency of transformation.

The fact that RecA does not play a major role in plasmid transformation raises the question if RecA threads are also formed during uptake of supercoiled plasmid DNA (which was taken as a source for oligomeric ssDNA). There was no visible change of pattern with regard to the formation of RecA threads after addition of plasmid DNA. Of the competent cells (∼20% of cells grown to competence), ∼65% showed RecA signals containing polar foci, ∼12% contained foci at various intracellular positions (other then the poles), and ∼23% showed GFP-RecA threads after addition of plasmid DNA (Figure 2D). Similarly, after addition of chromosomal DNA, ∼63% of the competent cells contained polar foci, ∼13% foci at other positions, and ∼24% formed threads (Figure 2B). Thus, formation of RecA threads is not specific for the kind of DNA substrate entering the cell during transformation.

### RecO assembles at a single cell pole in response to incoming plasmid or phage DNA

During DSB repair RecO is necessary to load RecA onto SsbA coated ssDNA [28]. Our genetic data show a RecR- and RecF-independent role for RecO during plasmid transformation (see Table 1). To analyze if RecO shows a particular pattern of localization after addition of DNA to competent cells, a functional RecO-YFP fusion was used (see Material and Methods). The same amount of chromosomal, plasmid or viral DNA was added in these experiments (0.1 µg/ml). Under this condition, transformation with chromosomal DNA was 200 to 1000 times more efficient (transformants per µg of DNA added) compared with plasmid transformation, although DNA concentrations curves for plasmid and chromosomal transformation revealed that both are first order processes (data not shown).

**Figure 3:**
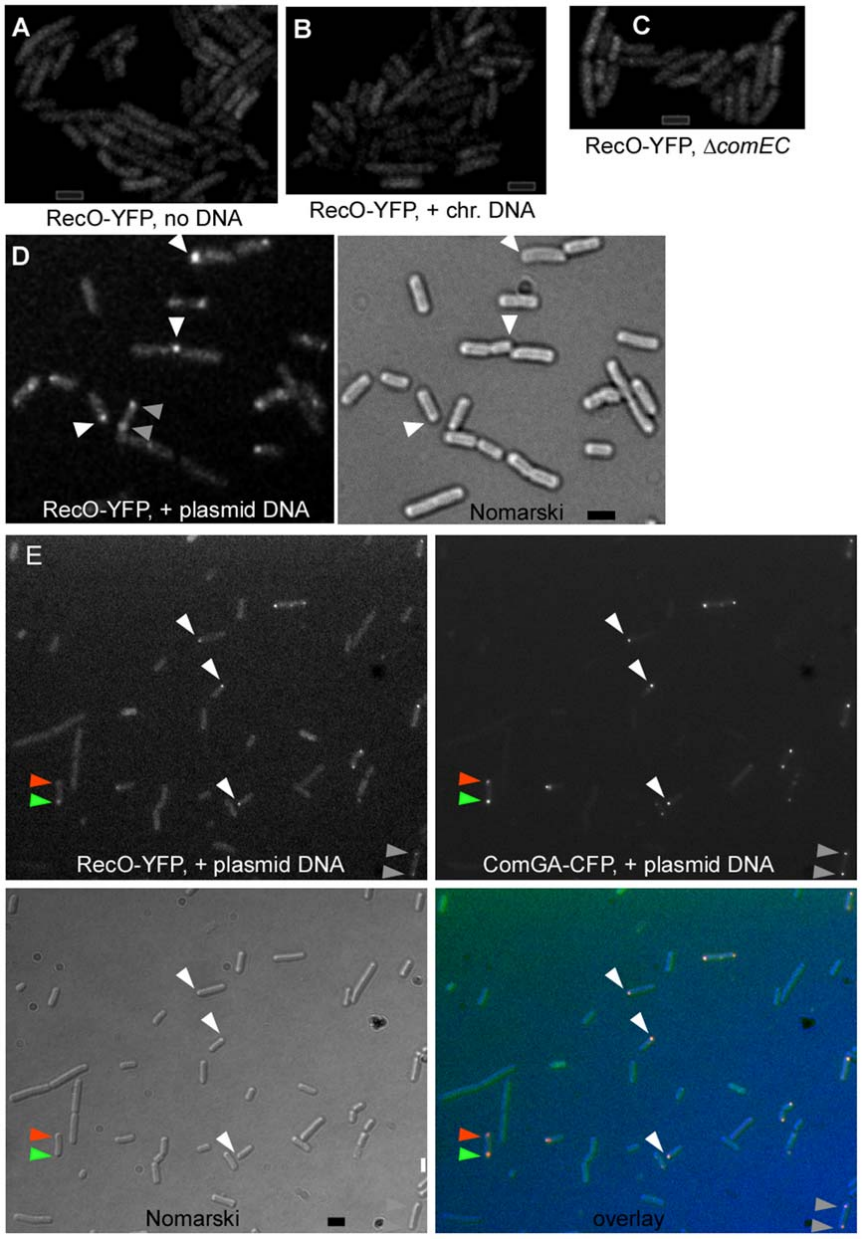
Fluorescence microscopy of cells grown to competence expressing RecO-YFP. (A) Fluorescence of RecO-YFP in wt competent cells without added DNA, (B) after addition of chromosomal DNA, (C) after addition of plasmid DNA in *comEC* mutant cells, and (D) after addition of plasmid DNA in wt cells, white triangles indicate single foci, grey triangles two polar foci. Note that RecO-YFP foci are overrepresented in this field, as only 17% of all cells analysed show detectable RecO-YFP foci. (E) Fluorescence of RecO-YFP and of ComGA-CFP in cells expressing both fusions, after addition of plasmid DNA. White triangles show single polar foci, grey triangles two polar foci, and orange triangle indicates a cell having two ComGA foci, a stronger one colocalizing with a RecO-YFP focus (green triangle) and a weaker one where no RecO-YFP focus is detectable. Black bar 2 µm.

RecO-YFP was dispersed throughout cells grown to competence (Figure 3A) and addition of chromosomal DNA did not alter this pattern: none of 600 cells analyzed contained visible RecO-YFP foci (Figure 3B). Strikingly, RecO-YFP formed a single focus at one cell pole in 5% of the cells grown to competence, as soon as 5 min after addition of supercoiled plasmid DNA. Up to 16.9% (102 out of 600 cells) of the cells showed a polar focus or two polar foci 30 min after addition of supercoiled plasmid DNA (Figure 3D, 26% of the cells having RecO foci contained two polar foci, indicated by grey triangles). These findings reveal a striking substrate- and time-dependent recruitment of RecO to the DNA uptake machinery.

To verify, that the RecO foci correspond to sites where the competence machinery is present, we combined the RecO-YFP strain with a ComGA-CFP fusion. In ∼18% of cells grown to competence (99 out of 550 cells analyzed), RecO-YFP colocalized with ComGA to a single cell pole (in 72% of these cells) or to both cell poles (in 28% of these cells) (Figure 3E), or was present throughout the remaining cells. 2 cells out of 550 showed one polar ComGA-CFP focus but no RecO-YFP focus, and 3 cells had two polar ComGA-CFP foci, but only one RecO-YFP focus was present, colocalizing with one ComGA-CFP focus (Figure 3E, indicated by green and orange triangles). These data show that RecO foci largely colocalize with the DNA uptake machinery upon addition of plasmid DNA.

**Figure 4:**
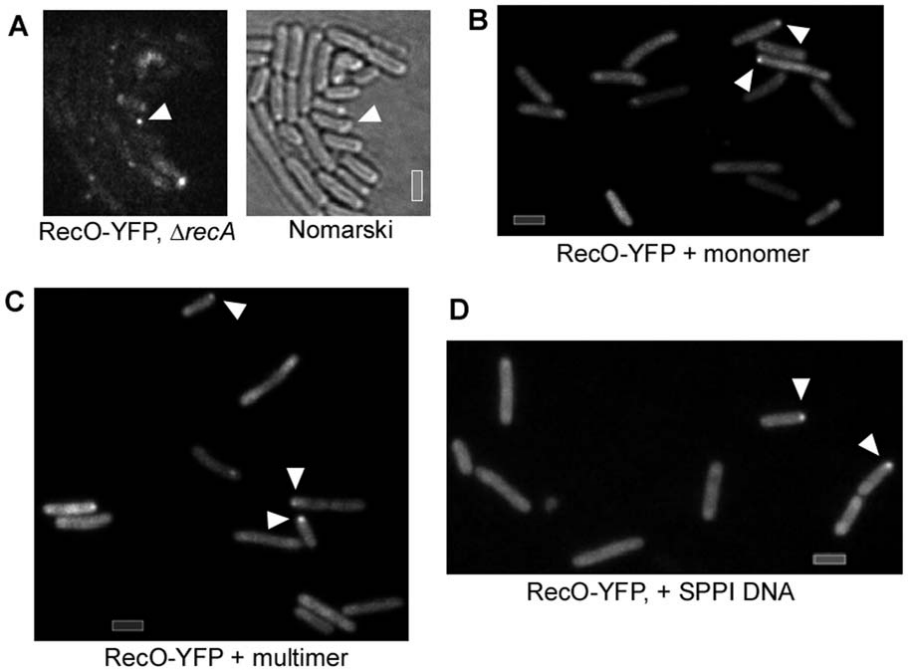
Microscopy of cells grown to competence expressing RecO-YFP after addition of various kinds of DNA. (A) Fluorescence of RecO-YFP in *recA* mutant cells plus plasmid DNA, (B) wt cells plus linearised monomeric plasmid DNA, (C) wt cells after addition of linearized trimeric and higher multimeric plasmid DNA, and (D) after addition of SPP1 phage DNA. Grey bars 2 µm.

Plasmid preparations from E. coli cells contain monomeric as well as multimeric plasmid forms. Only multimeric plasmid DNA has been shown to lead to transformation of B. subtilis cells [16],[33], so we generated monomeric as well as multimeric plasmid preparations. In contrast to transformation with chromosomal DNA, 8% of the cells grown to competence (i.e. 40% of all competent cells) showed RecO-YFP foci at the pole 30 min after addition of linearized monomeric plasmid DNA (Figure 4B, 32 foci in 400 cells). Similarly, addition of linearized dimeric plasmid DNA induced foci in 8.8% of the cells (data not shown). However, 30 min after addition of trimeric and higher multimeric plasmid DNA (all higher multimers were pooled because they could not be clearly separated), 13.5% of the cells (i.e. ∼68% of all competent cells) showed polar RecO-YFP foci (Figure 4C, 54 foci in 400 cells analysed). Thus, RecO-YFP foci are induced by monomeric or dimeric plasmid DNA, and significantly (2×2 chi^2^ value 6.3 with significance value of 0.012) increased in number after addition of multimeric plasmid DNA.

A major difference between plasmid and chromosomal transformation is the fact that upon addition of the same amount of DNA (as was done in these studies), the amount of identical DNA fragments taken up by a single competent cell is more than 1000-fold higher with plasmid than with chromosomal DNA. To test if a chromosomal DNA fragment with a size even smaller than monomeric plasmid DNA can also induce the formation of RecO-YFP foci, we generated a 3.5 kb DNA fragment carrying a tetracycline resistance gene flanked by 1 kb regions homologous to the chromosome on each side, which integrates into the non-essential ypbR locus by a double cross-over, in a RecA-dependent manner. As expected, the frequency of recombination was similar to that with plasmid DNA, i.e. lower than with chromosomal DNA. 10 to 30 min after the addition of this PCR-amplified DNA to cells grown to competence, 10.5% of the cells showed RecO-YFP foci (63% with one polar focus, 34% with two polar foci, and 4% with three foci, 470 cells analysed, data not shown), showing that also small dsDNA fragments can efficiently induce the formation of RecO-YFP foci.

We also investigated the genetic requirements for the recruitment of RecO to the cell pole. The formation of RecO-YFP foci in response to plasmid DNA was abolished in *comK* mutant cells (data not shown) and in mutant cells lacking the DNA uptake channel ComEC (Figure 3C). These experiments support the idea that RecO assembles at the pole due to incoming ssDNA with self-annealing potential transported through ComEC.

To address the question whether polar localization of RecO after addition of plasmid DNA depends on other recombination proteins, we moved the *recO-yfp* fusion into a Δ*recN* strain or we placed the Δ*recA* mutation into a *recO-yfp* strain. The formation of RecO-YFP foci was somewhat reduced in Δ*recN* cells (9.5%, 62 foci in 650 cells analyzed) (data not shown), but remained fairly constant in Δ*recA* cells (16.3%, 106 foci/650 cells) upon addition of multimeric plasmid DNA (Figure 4A). These data suggest that during plasmid transformation, RecO acts independently of RecA, and is mildly influenced by RecN. Similarly, during DSB repair, the recruitment of RecO to DNA breaks is influenced by RecN, but is independent of RecA [23],[34].

We also investigated, if the addition of phage DNA (45.5 kb DNA from SPP1) might recruit RecO to the cell pole. Clear RecO-YFP foci were detected in 2% of cells grown to competence 30 min after addition of 0.1 µg/ml of SPP1 DNA (7 foci/340 cells analyzed, Figure 4D), showing that to a lesser degree than plasmid DNA, but in contrast to chromosomal DNA, uptake of phage DNA results in polar RecO accumulation.

DNA uptake occurs at an average speed of 80-nt/s [35], so several 1,000 bases can be present few minutes after addition of DNA within the cell to induce the formation of GFP-RecA threads and/or RecO-YFP foci, which are visible after only 5 min. Therefore, we tested if incoming ssDNA could be a substrate for RecO.

### RecO anneals ssDNA *in vitro*

**Figure 5:**
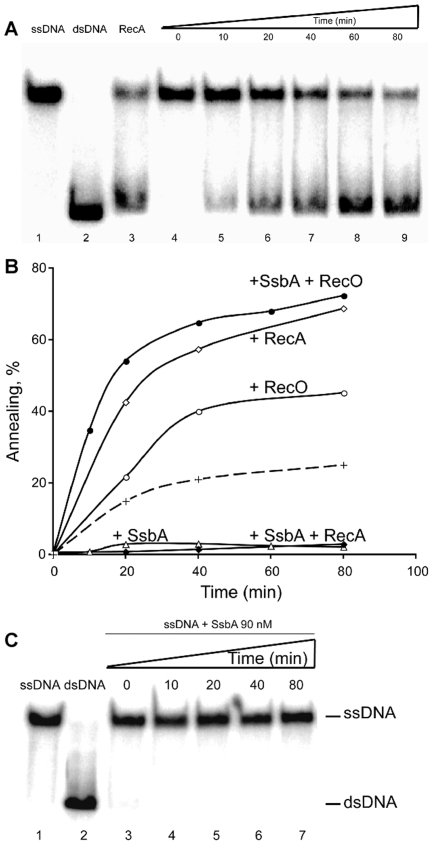
RecO anneals complementary ssDNA complexed with SsbA protein. (A) heat-denatured 440 nt long ssDNA (lane 1) was pre-incubated with SsbA (90 nM) for 10 min at 30°C, then RecO (500 nM) was added and the reaction incubated for a variable time (lanes 4 to 9). As controls, 440 bp dsDNA (lane 2) was heat-denatured (ssDNA, lane 1) and incubated with RecA (1.3 µM) in buffer A for 80 min at 30°C (lane 3). (B) Quantification of SsbA, RecA, and RecO annealing reactions. Heat-denatured 440 nt ssDNA was pre-incubated with 90 nM SsbA (+ SsbA, empty triangles). Then, 500 nM RecO (+ RecO, empty circle) or 1.3 µM RecA (+ RecA, empty diamonds) was added and the reaction incubated for variable time at 30°C. Heat-denatured 440 nt ssDNA was pre-incubated with 90 nM SsbA (+ SsbA) for 10 min. Then RecO (500 nM [+ RecO], filled circle) or RecA (1.3 µM [+ RecA], filled diamonds) was added and the reaction incubated for a variable time. The broken line denotes the rate of spontaneous annealing. The extent of DNA annealing is expressed as the percentage of the observed dsDNA signal relative to that of total DNA. (C) SsbA protein inhibits spontaneous annealing of complementary ssDNA. Heat-denatured 440 nt long ssDNA (lane 1) was pre-incubated with SsbA (90 nM) for variable times at 30°C (lanes 3 to 7). As control, native 440 bp dsDNA (lane 2) was incubated in buffer A for 80 min at 30°C (lane 2).

To investigate whether RecO can mediate the annealing of complementary ssDNA, we directly monitored DNA annealing of a heat-denatured 440-nt long DNA, or this substrate complexed with SsbA (Figure 5). RecO protein (at a ratio of 1 RecO/14-nt) enhanced the annealing of complementary ssDNA molecules, and similarly, RecA·dATP·Mg^2+^ (1 RecA monomer/3-nt) catalyzed the annealing of complementary ssDNA substrates (Figure 5A). Contrarily, SsbA, at a ratio of 1 tetramer/38-nt, inhibited the spontaneous annealing reaction (Figure 5C).

When the ssDNA substrate was pre-incubated with SsbA a different outcome was observed (Figure 5A and 5B). RecO protein (1 RecO/14-nt) efficiently promoted the annealing of complementary ssDNA substrates (Figure 5A, lanes 5-9). Similar results were observed if the RecO ratio was reduced to 1 RecO/28-nt (data not shown). However, RecA·dATP·Mg^2^ failed to catalyze the annealing of complementary ssDNA substrates complexed by SsbA (Figure 5B). Thus, RecO has potent ssDNA annealing activity *in vitro*, in the absence of any cofactor, and SsbA bound to ssDNA markedly stimulated this activity. However, SsbA exerted a negative effect on RecA·dATP·Mg^2+^-mediated DNA strand annealing.

### RecU is recruited to the competence pole, but dissipates after addition of DNA

**Figure 6:**
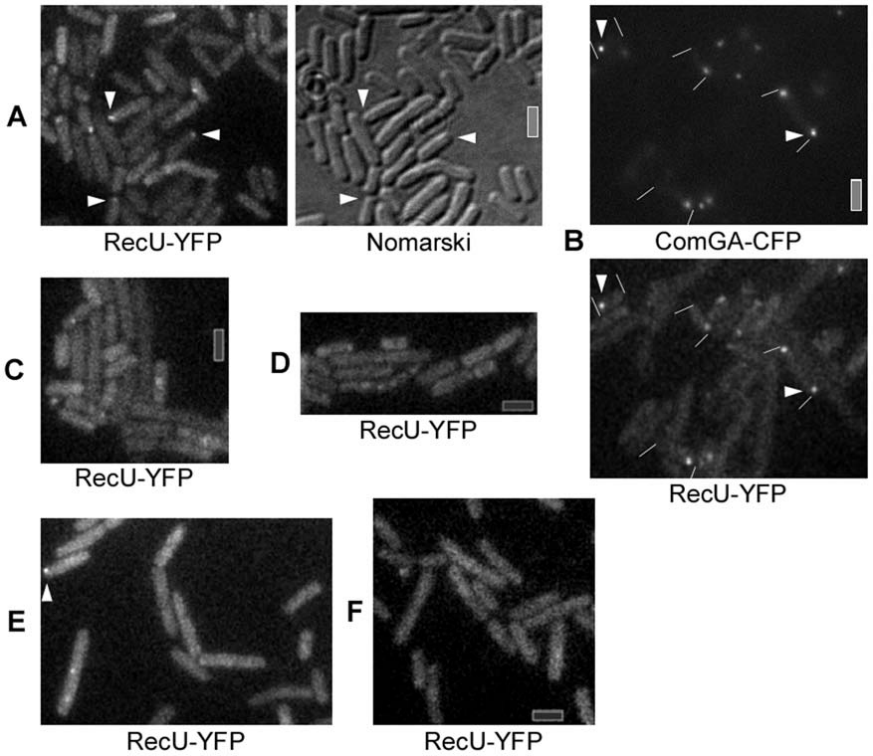
Fluorescence microscopy of cells grown to competence expressing RecU-YFP. (A) Fluorescence of RecU-YFP in wt cells, (B) wt cells also expressing ComGA-CFP, (C) in Δ*comK* mutant cells, (D) in Δ*recA* mutant cells, (E) in wt cells 15 min after addition of chromosomal DNA, and (F) in wt cells 30 min after addition of chromosomal DNA. White triangles indicate polar assemblies of RecU (and polar ComGA-CFP foci in panel B). Grey bars 2 µm.

We investigated the possibility of a colocalization of RecU and RecA during natural competence, because we showed that Δ*recU* cells are impaired in plasmid transformation whereas Δ*ruvAB* cells are not, and that RecU acts as a RecA modulator in a RuvAB-independent manner [36]. In cells grown to competence, we found that a functional RecU-YFP fusion (see Material and Methods) forms a focus at a single cell pole or two foci, each at a pole, in 18% (110 cells having foci/600 cells, with 27% of these having two foci) of cells grown to competence (Figure 6A). Polar RecU-YFP foci colocalized with polar ComGA-CFP foci in more than 95% of the cells containing foci (Figure 6B, 350 cells analyzed), showing that RecU assembles at the DNA uptake machinery in the absence of transforming DNA. This is consistent with the absence of polar RecU-YFP foci in *comK* mutant cells (Figure 6C). Interestingly, the formation of a RecU focus was also dependent on RecA protein. The number of cells containing polar RecU foci was reduced by 92% in the absence of RecA compared to wild type cells (>200 cells analyzed, Figure 6D), and the fluorescence intensity of the few foci was much lower than in wild type cells. This is consistent with the findings that RecU physically interacts with RecA and acts as a modulator of RecA [30],[36], and is involved in plasmid transformation and viral transfection (see Table 1). These observations markedly differ from RecU assembly during DNA DSB repair. Here, the accumulation of RecU-YFP foci, at late times after DSB induction, is strictly dependent on the presence of the RuvAB complex [25].

Strikingly, RecU-GFP foci dissipated from the pole after addition of any kind of DNA. Only 4% of all cells grown to competence contained polar (or any) RecU-GFP foci 15 min after addition of DNA (Figure 6E, 200 cells analyzed), while after 30 min, foci were not detectable in all 250 cells analyzed (Figure 6F). Like RecA (see above), RecU was statically located at the DNA uptake apparatus in the absence of external DNA (data not shown), but apparently changes its pattern of localization in response to incoming ssDNA.

## Discussion

Our work provides genetic and cell biological evidence that two different pathways operate during transformation with plasmid or with chromosomal DNA, and that proteins involved in DSB repair obtain different functional specificities during horizontal gene transfer. Using a prophage-free B. subtilis strain we have confirmed and extended previous data that in the absence of RecA, chromosomal transformation is abolished, whereas plasmid transformation operates normally. Conversely, the absence of RecO and RecU had little effect on transformation with chromosomal DNA, but severely impaired plasmid transformation [8,9,12,13, this work]. Interestingly, the absence of RecA suppresses the RecU requirement during plasmid transformation [30]. RecU has three activities: to cleave HJs, to catalyze annealing of complementary ssDNAs and to modulate RecA activity in vitro [27]. The recU71-encoded RecUR71A is proficient in strand annealing and HJ cleavage, but deficient in RecA interaction [30] and in plasmid transformation, suggesting that the main defect in the recU71 strain is the modulation of RecA, and that in the absence of RecU, the presence of RecA is counter productive for plasmid transformation.

**Figure 7:**
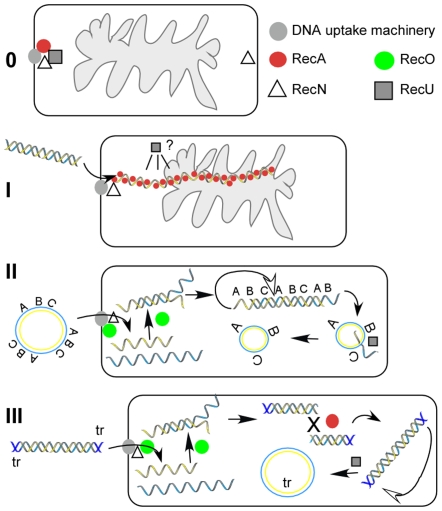
Model for events during transformation with different kinds of DNA. (0) Several recombination proteins accumulate at the polar DNA uptake machinery and RecN oscillates between the poles. (I) Upon addition of chromosomal DNA (and in fact any kind of DNA), RecN binds to incoming ssDNA, and RecA forms filamentous thread structures, which are thought to mediate recombination with a homologous region on the chromosome (indicated by gray cloud). In parallel, RecU loses its static position. Degradation of the displaced recipient DNA, and ligation of donor and recipient DNA generate the recombinant product. (II) RecO visibly accumulates at the DNA uptake machinery after addition of plasmid DNA. Oligomeric plasmid DNA is shown, indicated by the letters ABC, with one strand in blue and one in yellow. RecA also forms dynamic threads, which are not shown, because these are non-productive, RecU, which loses its static position, may modulate RecA. RecO anneals incoming ssDNA to dsDNA fragments, which can be assembled into circular plasmid DNA through intramolecular recombination, if internal homology is present (indicated by letters). (III) Viral ssDNA is converted into dsDNA fragments through recombination, and overlapping fragments need to recombine to generate a full-length phage DNA via both RecA- and RecO-mediated recombination. Recombination of terminal repeats (tr, dark blue) generates a circular phage molecule. Note that the chromosome is omitted from II and III.

In cells grown to competence, RecA, SsbB, DprA and CoiA (YjbF) are recruited to the DNA uptake machinery [6],[7],[18],[19], whereas RecN oscillates between the poles [19] (Figure 7-0). We show that in the absence of transforming DNA, RecU also accumulates at the competence pole (Figures 4 and 7-I), whereas RecO is dispersed throughout the competence cells. For the cytosolic proteins that are involved in the processing of internalized ssDNA, only the synthesis of RecA, SsbA, SsbB, DprA and CoiA is induced or increased during the state of competence [3],[4], while that of RecN, RecO and RecU is not. Therefore, the latter proteins gain novel functional specificity during horizontal gene transfer. Interestingly, the cytosolic recombination machinery responds differentially to the uptake of different forms of DNA. Upon addition of plasmid DNA, an accumulation of RecO at the cell pole, where the DNA uptake machinery assembles, was observed, but not after addition of chromosomal DNA (Figure 7-II). We show that RecO is able to efficiently anneal SsbA covered ssDNA in vitro, even at very low protein concentrations, and independent of a nucleotide cofactor. Because RecA is not required during plasmid transformation, it is clear that at least partly, RecO reassembles plasmid DNA from incoming ssDNA in vivo, which is then established as self-replicating unit.

### Functional requirements and spatial organization of transformation with homologous chromosomal DNA

In cells grown to competence, RecA and RecU are present at the DNA uptake machinery, and RecN oscillates between the poles [19, this work], whereas RecO is dispersed throughout the cytosol (Figure 7-0). Upon addition of any kind of DNA RecN localizes to the pole that contains the DNA uptake machinery (Figure 7-I), and RecA and RecU lose their static position at the cell pole (Figure 7-I). RecN specifically binds to the 3′-OH end of ssDNA in vitro and protects it from exonuclease attack [20],[21], but plays a minor role in transformation in the phage free strain background. Modulators of RecA must displace the single-stranded binding proteins (e.g., SsbA, SsbB) and help loading of RecA onto ssDNA, which has been demonstrated for DprA [22]. Thus, RecA binds to incoming ssDNA and forms threads that emanate from the uptake machinery [19]. Further investigating RecA thread formation, we found that RecA threads are highly dynamic structures that change their length and orientation within a 1 min time scale. Maximum extension of filaments was measured to be 0.6 (±0.2) µm/min, similar to the observed speed of spreading of RecA on dsDNA in vitro [32], suggesting that filament growth and shrinkage may be mediated by RecA coating of and dissociating from incoming ssDNA. These finding reinforce the idea that RecA threads are actively searching for homology of the incoming DNA with the recipient chromosome. RecA/donor ssDNA invade recipient duplex DNA, forming a D-loop structure. Interestingly, RecU also dissipated from the pole after addition of DNA (Figure 7-I). In agreement with the association at the cell pole of RecA and RecU, and their loss of this static position after DNA addition, both proteins have been shown to physically interact with each other [30]. Possibly, RecU protein tracks along with RecA threads that move away from the cell pole and may enhance RecA-mediated D-loop formation, which is consistent with data showing that RecU enhances RecA-promoted DNA strand invasion [27],[36]. However, D-loop intermediates cannot be resolved by the RecU HJ-resolvase in vitro [27]. From the results obtained we can also infer that in DNA transformation, 4-strand recombination intermediates (HJs) are not formed, because HJ resolution through RecU is not required for transformation and in the absence of the RuvAB translocase, chromosomal transformation is also not affected. Clearly, an as yet unknown D-loop resolvase and a ligase are needed to mediate full incorporation of taken up homologous DNA (Figure 1A). Replication of the generated heteroduplex will generate one transformed daughter cell.

### Establishment of non-homologous self-replicative DNA within competent cells

The uptake of plasmid DNA follows the same pathway and kinetics through the membrane via the uptake machinery than that of chromosomal DNA [1],[2]. In the presence of internalized plasmid ssDNA, RecA also forms threads, showing that RecA is loaded onto any kind of incoming ssDNA. However, the RecA threads are unproductive if the incoming ssDNA shares no significant homology (larger than 50-nt) with recipient DNA, as is the case for plasmid DNA. In the absence of sufficient homology, RecU may promote the disassembly of RecA from the taken up ssDNA, which should then become coated by a single-strand binding protein (e.g., SsbA, SsbB, DprA). Strikingly, incoming plasmid DNA triggers the recruitment of RecO to the competence machinery. Possibly, RecO accumulates at the competence pole through direct protein-protein interaction with SsbA bound to incoming ssDNA, consistent with *in vitro* data showing that RecO physically interacts with SsbA [28]. All other proteins induced by competence possibly covering the entering ssDNA (namely, DprA/Smf and SsbB) are present at the competence pole even in the absence of incoming DNA, and thus cannot cause the switch of recruitment of RecO in response to incoming plasmid DNA. It is also possible that an unknown factor causes the accumulation of RecO at the competence machinery. However, we propose that RecO accumulates due to its annealing activity. Taken up ssDNA must anneal to form dsDNA fragments for plasmid establishment (Figure 7-II). The likelihood that both complementary Watson and Crick strands are taken up is several orders of magnitude higher during uptake of a similar amount of plasmid DNA (∼15 kb) than of chromosomal DNA (4,200 kb). RecO is a dimer in solution and binds cooperatively to ssDNA with high affinity [28], but has drastically lower affinity to dsDNA (C.M. and B.C., unpublished). Additionally, RecO has much higher annealing activity with complementary ssDNA strands complexed with SsbA than RecA *in vitro* (Figure 5), therefore, incoming plasmid DNA provides a high amount of substrate for RecO. Interestingly, the number of competent cells having visible polar RecO-YFP foci was higher after addition of oligomeric DNA compared with monomeric DNA. Repetitive (homologous) sequences increase the annealing potential, supporting the view that RecO accumulates due to its activity in strand annealing. To further test this idea, we investigated the effect of the addition to cells grown to competence of a 3.5 kb construct that integrates into the recipient chromosome by a double-cross over event (in a RecA-dependent manner). RecO-YFP foci accumulated at the cell poles in a number of cells in between that for monomeric or for multimeric plasmid DNA, further supporting the notion that RecO accumulates at the DNA import machinery due to a high amount of complementary DNA strands.

RecU has also been shown to possess strand-annealing activity *in vitro* [27],[30], and is also important for plasmid transformation. We propose that the main role of RecU in plasmid transformation is to down regulate RecA activity rather than to mediate strand annealing. Indeed, it was shown that the RecU requirement during plasmid transformation can be overcome by deleting *recA* [30]. Electron microscopy analyses revealed that: (i) purified RecU does not promote RecA disassembly from ssDNA, but discrete RecU blobs embedded in a RecA nucleoprotein filament reduces RecA dynamic assembly, and (ii) RecU alone does not polymerizes onto ssDNA [36]. Down-regulation of RecA may be important for efficient plasmid DNA establishment, because RecA is inefficient in catalyzing DNA strand invasion on linear dsDNA, in contrast to supercoiled DNA, and may hinder the formation of circular plasmid dsDNA.

We also found a novel function for Ku protein during transformation with plasmid (but not with chromosomal) DNA. Ku might protect DNA ends that arise during DNA annealing (Figure 7-II) from nuclease attack. A Ku-GFP fusion was dispersed throughout cells grown to competence (data not shown), and its expression was markedly increased during competence, compared with growing cells, supporting an important function for Ku during plasmid horizontal gene transfer.

As a second step, annealed duplex and partially duplex DNA must circularize to establish a plasmid molecule (Figure 7-II). As previously documented for RecO*_Eco_* [37], we propose that RecO promotes the annealing of a homologous region of the same molecule complexed by SsbA, to generate a circular molecule (Figure 7-II). This intramolecular recombination only generates unit-length molecules if the substrate has internal redundancy. Consistent with this idea, it has been shown that plasmid transformation can be achieved with single trimer or higher multimer molecules, but not with monomeric plasmid DNA [16],[33]. Once an oligomeric plasmid molecule is circularized and replicated, the host-encoded resolution system should resolve it to monomeric plasmid DNA. These proposed steps provide an economic avenue to reassemble plasmid DNA with few proteins, and independently of RecA.

### Transfection of viral SPP1 DNA follows a mixed route

During phage transfection with mature viral DNA, the functions of RecA, RecO, RecU and Ku are required. Unlike chromosomal and plasmid transformation, viral transfection requires the recombination of 2 to 4 DNA molecules to yield a 45.5 kb viral DNA [38]. Phage DNA must first anneal to form dsDNA segments, similar to plasmid transformation (Figure 7-III). Interestingly, RecO also accumulated at the pole after addition of SPP1 DNA, but in fewer cells compared with plasmid DNA, most likely because the different segments of the viral DNA have less annealing potential than the oligomeric plasmid DNA. As a second step, overlapping fragments must recombine to form a full-length phage DNA molecule. This intermolecular recombination event requires RecA and the phage recombination machinery (e.g. strand annealing protein, G*35*P, and 5′to 3′exonuclease G*34.1*) [39],[40]. The terminal repeat regions (Figure 1C) then recombine, perhaps via single-strand annealing, to generate a circular phage molecule that can replicate. Thus, genetic transformation follows different pathways, which in the case of phage transfection contains steps from both chromosomal and plasmid transformation.

Contrarily to natural transformation, DSB repair appears to follow one discrete avenue. Cell biological experiments have documented that upon induction of DSBs, recombination proteins are recruited to the DNA damage site on the nucleoid in a relatively fine tuned temporal order (setting up a so-called repair center), with RecN assembling first, followed by RecO (which is important for loading of RecA onto SsbA-coated ssDNA), and RecA itself, and later RecF and RecU [15],[23],[25],[41]. Thus, the recombination machinery can switch between DNA repair and incorporation or establishment of foreign DNA within the cell, and assembles differentially according to the different DNA substrates taken up from the environment.

## Materials and Methods

### Bacterial strains, plasmids, and reagents

*E. coli* XL1-Blue (Stratagene) was grown in Luria–Bertani (LB) rich medium supplemented with 50 µg/ml ampicillin where appropriate. *B. subtilis* strains were grown in LB rich medium at 37°C, or in defined minimal medium for microscopy. The strains used in this study are described in Table 2.

**Table 2:** Strains used in this study.

Strains	Genotypes	References
SB19^a^	Prototoph	wt
BG214^b^	*trpC*2 *metB*5 *amyE sigB*37 *xre*1 *att* ^SPβ^ *att* ^ICE*Bs*1^	Wt
BG129	*recF15*	[9]
BG277	*recN::cat* (Δ*recN*)	[45]
BG439	*recO::cat* (Δ*recO*)	[12]
BG190	*recA::cat* (Δ*recA*)	[43]
BG427	*recU::cat* (Δ*recU*)	[13]
BG1021	*recU71*	[30]
BG809	*ykoV::cat* (Δ*ku*)	[46]
BG128	*recR::cat* (Δ*recR*)	[47]
BG697	*ruvAB::cat* (Δ*ruvAB*)	[25]
PY79^c^	Prototoph	wt
DK4	*recF-yfp*	This work
DK2	*recO-yfp*	This work
DK80	*recO-yfp,* Δ*comK*	This work
DK81	*recO-yfp,* Δ*comGA*	This work
DK82	*recO-yfp,* Δ*recA*	This work
DK83	*recO-yfp,* Δ*comEC*	This work
DK26	*recO-yfp,* Δ*recN*	This work
DK84	*recO-yfp, comGA-cfp*	This work
DK53	*recU-yfp*	This work
DK86	*recU-yfp,* Δ*comK*	This work
DK87	*recU-yfp,* Δ*comG*	This work
DK88	*recU-yfp, comGA-cfp*	This work
DK89	*recU-yfp,* Δ*recA*	This work
ST01	*ykoV-yf*	This work

a SB19 is cured of SPβ phage and non-inducible for PBSX prophage, and lacks the ICE*Bs*1 transposon.

b Strains of the BG series are isogenic with BG214 (cured of SPβ, non-inducible for PBSX, and lacks the ICE*Bs*1).

c Strains of the DK and ST series are isogenic with strain PY79.

### Construction of vectors and strains

The *recN*, *recO*, or *recU* genes were fused at their 3′- end with the *cfp* or the *yfp* gene and the fused gene was integrated into the chromosome by single crossover integration to replace the wt gene as previously described [23],[25]. Thereby the *recN-*, *recO-* or *recU-yfp* gene fusions were present at the native locus and transcribed from their native promoters, which are not induced during competence development [3],[4]. RecN-YFP, RecO-YFP or RecU-YFP expressing cells were as resistant to methylmethane sulfonate and to MMC as the wt strain [23],[25] and had transformation efficiencies like wt cells (data not shown), showing that the fusions were fully functional. The *recA* gene was fused at the 5′-end to the *gfp* gene, the fused gene was placed under the control of the xylose inducible promoter and ectopically integrated into the *amy* locus. Then the wt *recA* gene was deleted [19]. The resulting GFP-RecA strain was as sensitive to MMC as the wt strain [23],[25] and had transformation efficiencies like wt cells (data not shown), showing that the fusion is fully active.

To move the *recN-cfp*, *recO-yfp* or *recU-yfp* fusion in different mutant backgrounds, transformation with chromosomal DNA was used (Table 2). For the colocalization experiments, strain DK2 (*recO-yfp*) was transformed with chromosomal DNA from *comGA-cfp*, generating strain DK84. For the colocalization experiments, strain DK53 (*recU-yfp*) was transformed with chromosomal DNA from *comGA-cfp*, generating strain DK88. A Ku-YFP fusion was constructed by cloning the PCR amplified 3′ end (500 bp) of the *ykoV* gene into pSG1164, which was integrated into the chromosome via single crossover, such that the expression of the downstream *ykoU* gene was driven by the xylose promotor. The Ku-YFP fusion was fully functional, as all above stated YFP fusions.

### Transformation assays

Competent cultures were grown as described previously [42]. Competent *B. subtilis* cells were transformed with pUB110 plasmid DNA, chromosomal DNA from a *met* ^+^ strain (SB19 DNA) or bacteriophage SPP1 DNA. The yield of kanamycin-resistant transformants (plasmid transformation), *met* ^+^ transformants (chromosomal transformation), and SPP1 transfectants was corrected for DNA uptake (assayed through the determination of uptake of radioactively labeled DNA into cells grown to competence through DNaseI degradation of the labeled DNA) and for cell viability (viability counts), and the values obtained were normalized relative to that of the *rec* ^+^ strain, which is taken as 100 [9],[43].

### Complementary ssDNA annealing assays

RecO, RecA and SsbA proteins were purified as previously described [28],[36]. To determine whether RecO anneals complementary ssDNA coated with SsbA, buffer A (50 mM Tris-HCl [pH 7.5], 1 mM DTT, 80 mM NaCl, 2 mM EDTA, 50 µg/ml bovine serum albumine [BSA], 5% glycerol) was used, whereas in RecA-SsbA reactions, buffer B (50 mM Tris-HCl [pH 7.5], 1 mM DTT, 40 mM NaCl, 10 mM magnesium acetate, 2 mM dATP, 50 mg/ml BSA, 5% glycerol) was used, and the DNA complexes were monitored upon deproteination. The heat-denatured 440-nt ssDNA (7 mM in nt, pGEM-3Zf(+) *Eco*RI[5]-*Afl*III[445] DNA fragment) when indicated was pre-incubated with SsbA (90 nM) for 10 min at 30°C. Then RecO (500 nM) or RecA (1.3 mM) was added and the reaction incubated by a variable time. The samples were deproteinized as described [27], separated in a native (n) 6% polyacrylamide gel electrophoresis (nPAGE), and the gels dried prior to autoradiography and quantification as previously described [27].

### Image acquisition

Fluorescence microscopy was performed on an Olympus AX70 microscope. Cells were mounted on agarose pads containing S750 growth medium on object slides. Images were acquired with a digital MicroMax CCD camera; signal intensities were measured using the Metaview program. DNA was stained with 4′,6-diamidino-2-phenylindole (DAPI; final concentration 0.2 ng/ml), and membranes were stained with FM4-64 (final concentration 1 nM). Chromosomal DNA (from *B. subtilis* or *E. coli*) or plasmid DNA (various replicative plasmids) were added to 100 µl of cells grown to competence, resulting in a final concentration of 0.1 µg/ml of DNA. For purification of mono or multimeric plasmid DNA, plasmid pDG148 was digested with *Eco*RI, and was purified after agarose gel electrophoresis. Monomeric plasmid DNA was incubated with LrpC protein and DNA ligase to enhance formation of dimeric and higher multimeric DNA [44] The ligated DNA was purified from low melting agarose using phenol-chloroform extraction.

## Supporting Information

Video S1 Time lapse microscopy (1 min intervals) of a cell grown to competence containing a GFP-RecA thread 15 min after addition of chromosomal DNA. 6 frames/s. First frame shows membrane staining of the cells. Movie corresponds to Figure 1C left panel.
(0.08 MB MOV)

Video S2 Time lapse microscopy (1 min intervals) of a cell grown to competence containing a GFP-RecA thread 15 min after addition of chromosomal DNA. 6 frames/s. Movie corresponds to Figure 1C right panel.
(0.04 MB MOV)

Video S3 Time lapse microscopy (1 min intervals) of a cell grown to competence containing a GFP-RecA thread 15 min after addition of chromosomal DNA.
(0.08 MB MOV)

Video S4 Time lapse microscopy (1 min intervals) of two cells grown to competence containing a GFP-RecA thread 15 min after addition of chromosomal DNA. First frame shows membrane staining of the cells.
(0.14 MB MOV)
